# Critical Assessment of the Neurological Complications during High-Risk Anesthesia Procedures

**Published:** 2024-06-06

**Authors:** Fihr Chaudhary, Zubair Ahmed, Devendra K Agrawal

**Affiliations:** Department of Translational Research, College of the Osteopathic Medicine of the Pacific, Western University of Health Sciences, Pomona, California USA

**Keywords:** Analgesics, Anesthesia, Delirium, Neurological complications, Postoperative cognitive decline, Postoperative visual loss, Spinal cord ischemia

## Abstract

Damage to the peripheral and central nervous systems is frequently irreversible. Surgically induced neurological damage and anesthesia may result in catastrophic situations for patients and their families. The incidence of significant neurological complications during the perioperative period is examined in this article. In contrast to other organs like the kidney, heart, liver, lungs, and skeletal system, native neurological function cannot be replaced with artificial parts or devices soon. Ignoring brain function during the perioperative period has been a systemic problem in anesthesiology, even though the central and peripheral nervous systems are crucial. This bold claim is intended to draw attention to the fact that, unlike the circulatory and respiratory systems, which have been routinely monitored for decades, the brain and other neural structures do not have a standard monitoring during surgery and anesthesia. Given that the brain and spinal cord are the principal therapeutic targets of analgesics and anesthetics, this deficiency in clinical care is even more alarming. Organs that are notoriously hard to repair or replace after damage have, up until now, received comparatively little attention. In this article, a succinct overview of five neurological complications associated with surgery and anesthesia is presented. After critically reviewing the literature on the subject, the article is focused to common (delirium), controversial (postoperative cognitive decline), and potentially catastrophic (stroke, spinal cord ischemia, or postoperative visual loss) adverse events in the neurological surgery setting. The findings will increase awareness of major neurological complications to the involved surgical and anesthesia team and enhance preventive and treatment strategies during the perioperative period.

## Common Neurological Complications Related to Anesthesia

### 1.1 Delirium

Delirium is a temporary and variable neurological condition that indicates a deviation from normal cognitive functioning. It is distinguished by the key characteristics of inattention and disorganized thinking [1,2]. Delirium is widely recognized as a significant postoperative complication due to its prevalence, impacting approximately 70% of patients aged 60 years and above who undergo major inpatient surgeries. Moreover, it is linked to unfavorable consequences such as mortality, enduring cognitive deterioration, and extended periods of intensive care and hospitalization [3]. The occurrence of delirium or agitation during recovery from general anesthesia is a common phenomenon, particularly in pediatric patients [4]. However, it will not be addressed in the present section. Most of the discussion will be directed towards postoperative delirium due to its notable importance as a complication linked to heightened morbidity and mortality rates. Postoperative delirium is frequently observed in a significant number of patients, serving as an indicator of brain susceptibility. Its presence implies the potential presence of an underlying neurological disorder, such as early or preclinical dementia [5]. Delirium, although its frequent occurrence and significant consequences, is often not identified due to its manifestation of a hypoactive rather than hyperactive phenotype [6]. In addition, in the absence of focused inquiry, patients may exhibit a normal appearance or even a mild sense of lethargy. Reliable and user-friendly diagnostic methods have been developed to facilitate the diagnosis of delirium.

Historically, anesthetists have not prioritized the management of delirium due to its tendency to develop when patients are no longer under their direct supervision. Delirium is a distressing condition that affects both patients and their families. It poses a daunting challenge for physicians, as there are currently no recognized treatments to reduce its occurrence or shorten its length. The prevention and treatment of delirium pose challenges due to the involvement of various pathogenic processes, such as imbalances in neurotransmitters, neuroinflammation, malfunction of endothelial cells, poor oxidative metabolism, and changes in the availability of large neutral amino acids [7,8]. Given the intricate nature of the issue, it is improbable that any singular intervention will serve as a universal solution. However, it is crucial to address and mitigate significant risk factors associated with delirium. Some of these symptoms include pain, difficulty sleeping, impaired sensation, loneliness, lack of natural light, infections, withdrawal syndromes, low blood volume, need for a blood transfusion, abnormal electrolytes or acid-base balance, hypoxia, abnormal body temperature, seizures, and malfunctions of the endocrine system [9]. Several medications frequently administered during the perioperative phase, including atropine, antihistamines, corticosteroids, benzodiazepines, propofol, and opioids, have the potential to induce delirium and should be reduced in patients who are susceptible to this condition [10]. Considering the prevalence of postoperative delirium, any intervention aimed at its prevention or mitigation would provide significant clinical implications. Randomized controlled trials have recently provided evidence that using a processed electroencephalogram to guide the administration of both complete anesthesia and volatile-based general anesthetics could potentially reduce the occurrence of postoperative delirium [11]. One possible explanation for this phenomenon is that a processed electroencephalogram helps to avoid administering an excessive amount of anesthesia to patients who are more susceptible to complications. Nevertheless, if a slightly more profound level of anesthesia elevates the likelihood of postoperative delirium in susceptible surgical patients, it is anticipated that regional anesthesia would be linked to a much-reduced occurrence of postoperative delirium compared to general anesthesia. The results of a meta-analysis of small trials that randomly assigned surgical patients to either regional anesthesia (with light sedation) or general anesthesia revealed that there was no significant association between general anesthesia and an increased risk of delirium (odds ratio, 0.88; 95% confidence range, 0.51-1.51) [12]. The observed paradox necessitates additional investigation by a comprehensive and practical clinical investigation.

### 1.2 Postoperative cognitive decline

Postoperative cognitive dysfunction (POCD) is a significant issue for older surgery patients and their families. Recent investigations have confirmed that a significant proportion, up to 50%, of older people who undergo both cardiac and non-cardiac surgery continue to experience persistent POCD [13]. POCD is a contentious diagnosis that is not explicitly outlined in the Diagnostic and Statistical Manual of Mental Disorders. Moreover, it is worth noting that there is currently no defined International Classification of Disease Code for POCD. The postoperative cognitive deficit disorder is a subtle and often temporary decrease in cognitive function that can only be identified by specific neuropsychological testing and a comparison with preoperative cognitive abilities. Studies have shown that elderly patients who undergo major surgery often experience POCD for a period of weeks to months. Specifically, 10% of patients over the age of 60 years still experience POCD three months following the procedure [14]. Early POCD has significant adverse effects on patients and their families, including causing delays in returning to work, being connected to higher death rates [15], and prematurely leaving the employment [16]. Nevertheless, it remains uncertain if early POCD often indicates patient vulnerability. Furthermore, when studies have tracked patients over an extended period, their cognitive progressions have resembled those of age- and disease-matched individuals [17-19]. This finding provides evidence in favor of the frailty or vulnerability theory, as it demonstrates that the occurrence of cognitive deterioration is consistent after 3 months following major surgery under general anesthesia, as well as after coronary angioplasty without surgery or under general anesthesia [14]. Recent findings from a Dutch multicenter experiment may cast doubt on long-held beliefs about POCD following heart surgery [20]. The present study employed a randomized design to allocate a total of 280 patients into two groups: percutaneous coronary intervention, which involved no surgical procedures or general anesthesia, and off-pump coronary artery bypass grafting. The cognitive function of these patients was thoroughly evaluated using a comprehensive set of nine neuropsychological tests. At the 7.5-year follow-up, the study revealed that the surgical group had comparable enhancements in cognitive function to the non-surgical group [20]. Given that significant cognitive decline is a common occurrence with aging, even in individuals as young as 45 years old, it is not surprising that studies lacking suitable age- and disease-matched controls have observed cognitive deterioration in older patients with different health conditions in the intermediate to long-term postoperative period. Several studies in this field have encountered significant constraints, such as the absence of suitably matched non-surgical controls, lack of data on preoperative cognitive trajectories, suboptimal statistical methods, and failure to consider cognitive outcomes in relation to the success of surgery and the overall postoperative course [21]. Take into consideration a hypothetical scenario involving an elderly individual with notable vascular disease, exhibiting cognitive decline prior to undergoing surgery, experiencing complications such as hypotension and substantial blood component transfusion, encountering a challenging postoperative recovery in the intensive care unit characterized by delirium, subclinical stroke, wound infection, and renal dysfunction, and enduring chronic pain resulting from surgical incisions. It would not be surprising if this hypothetical patient were to suffer from chronic POCD. Patients are likely to experience cognitive impairment in the early postoperative period due to the cognitive burden caused by pain, inflammation, and acute illness [22]. This cognitive impairment might be likened to sickness behavior. Nevertheless, following the recuperation process following surgical procedures, patients may revert to their anticipated cognitive trajectories, which are derived from their preoperative trajectories. Individuals who exhibit enhancement in their overall well-being in comparison to their initial state before surgery may also encounter a relative improvement in cognitive function. This phenomenon may arise when surgical intervention leads to a reduction in pain (e.g., from angina pectoris), a decrease in inflammation (e.g., from arthritis), an increase in cerebral blood flow, and an improvement in everyday functioning. The concept of cognitive improvement or decline following surgery is a subject of debate. However, it aligns with findings that demonstrate the brain's capacity for plasticity throughout an individual's lifespan. Additionally, initial studies have indicated that successful surgical procedures, such as back surgery, joint surgery, carotid surgery, cardiac surgery, and ventricular assist device surgery, may result in increased brain volumes or enhanced cognitive performance [23].

### 1.3 Stroke

A perioperative stroke is a type of cerebral infarction caused by either ischemia or hemorrhage that happens during or after surgery. The postoperative period for this type of stroke can be up to 30 days. The occurrence of perioperative stroke is most observed in cases of cardiac surgery and carotid endarterectomy, with an incidence rate ranging from 4% to 5%. In their study, Bucerius et al. [24] examined a cohort of 16,184 consecutive patients who underwent cardiac surgery. The researchers observed a perioperative stroke incidence rate of 4.8%, with double-valve surgery being linked to a risk of 10%. A total of 3526 patients who underwent carotid surgery under either general anesthesia or local anesthesia with sedation were examined in the multicenter GALA experiment [25]. The incidence of stroke was found to be 4% in all groups, indicating that general anesthesia does not provide a significant risk for perioperative stroke, particularly in the case of carotid endarterectomy. The prevalence of stroke among individuals who have undergone non-cardiac, non-vascular, and non-neurological surgical procedures is notably reduced. In their investigation, Mashour et al. [26] utilized the National Surgical Quality Improvement Program (NSQIP) database of the American College of Surgeons. A total of 500,000 patients who underwent various procedures were examined, and it was shown that the occurrence of perioperative stroke was 0.1%. However, when five or more risk factors were present, the incidence increased to 2%. In their study, Sharifpour et al. [27] utilized the NSQIP database to analyze a sample of 38,000 patients who underwent non-carotid vascular surgery. Their findings revealed an incidence rate of 0.6%. It is imperative to acknowledge that the data pertain to the occurrences of overt stroke, specifically stroke characterized by a clinically evident neurological impairment. The NeuroVISION experiment, which involved noncardiac surgery in patients with cardiovascular risk factors, has provided initial findings indicating that the occurrence of hidden stroke, defined as the absence of apparent deficit, is estimated to be 10%. This diagnosis is made using magnetic resonance imaging during the postoperative phase [28]. If substantiated by a broader trial, this notable observation could have significant ramifications for the investigation and mitigation of perioperative stroke following non-cardiac surgical procedures.

### 1.4 Spinal cord ischemia

Spinal cord ischemia is a severe complication that can occur because of surgical intervention aimed at repairing thoracoabdominal aneurysms and dissections. The reported occurrence of spinal cord injury (SCI) exhibits variability, which can be attributed to the heterogeneity of patient cohorts in terms of aneurysm type, preventive measures, and surgical approaches. In the past, the occurrence of SCI has been shown to range from 5% to 40% [29]. However, more recent research indicate that the prevalence is normally about 10% [30]. Immediate-onset SCI can occur when the blood supply to the spinal cord is interrupted during surgery. It is possible for a spinal cord infarction to be many hours old when it is first detected. The anterior spinal cord receives its native blood supply from dual sources, namely the singular anterior spinal artery and many segmental radicular (intercostal) tributaries. The interruption of blood flow through the intercostal arteries can result in acute SCI due to several procedures, such as collateral artery ligation during surgical dissection, aortic cross-clamp application, and collateral artery covering by an endovascular stent. Postoperative collateral thrombosis or a reduction in spinal cord perfusion pressure, such as hypotension or increased cerebrospinal fluid (CSF) pressure due to ischemia-reperfusion, or both, can contribute to the occurrence of delayed-onset SCI. The primary determinant of SCI is the precise position and size of the aortic aneurysm. Conrad et al. (31) conducted a study examining the association between the occurrence of SCI and the specific site of the aneurysm. Their findings revealed a declining incidence as the Crawford types I and II (originating from the upper thoracic region) progressed to type III (originating from the mid-thoracic region) and finally type IV (originating from the distal thoracic region). Additional risk factors that should be considered include the duration of cross-clamp duration, particularly when distal reperfusion techniques are not employed, emergency surgical procedures, occurrences of aortic rupture or dissection, and the potential presence of intraoperative hypotension. The identification and subsequent reimplantation of non-selected segmental intercostal arteries do not provide a definitive reduction in the risk of SCI, but it may offer potential advantages in cases where substantial aortic replacement is required. The occurrence of SCI in thoracic endovascular aneurysm repair may be comparatively lower, potentially due to a reduced number of risk factors connected with the surgery. This is even though the operation involves the coverage of many segmental arteries, and this further indicates a diverse range of SCI risk factors for each individual patient. Patients undergoing endovascular repair may face an increased risk of SCI if they have a history of infrarenal abdominal aortic aneurysm repair or internal iliac artery blockages, which can lead to reduced contributions to collateral flow.

### 1.5 Postoperative visual loss

Postoperative visual loss is an infrequent yet severe neurological consequence that can arise from elective surgical procedures. It can be attributed to various factors such as central retinal artery blockage, cortical blindness, and ischemic optic neuropathy (ION). Shen et al. (32) conducted a study utilizing the Nationwide Inpatient Sample database in the United States to determine the overall occurrence of posterior occlusion of the vertebral column (POVL). The findings revealed that heart surgery (0.09%) and posterior spine surgery (0.03%) were found to be linked to the highest risk of POVL. In their study, Patil and colleagues [33] utilized the identical database but applied distinct inclusion criteria. They observed a 0.09% occurrence of POVL in spine surgery, with 0.02% of this occurrence being attributed to ION. In their study, Stevens et al. (1996) observed a significantly elevated occurrence of postoperative lumbar vertebral stenosis following spinal surgery at three different institutions. The incidence rates were 0.2% for all causes and 0.1% specifically for ION. The variations in the occurrence are likely attributable to the database employed and the criteria for inclusion. Overall, the data indicate that heart surgery and posterior spine surgery are the types of surgeries with the highest risk for POVL and ION. However, it is important to note that this complication is not exclusive to these specific surgeries.

#### What are high-risk surgeries?

Following are various surgical procedures with possible significant effect on hemodynamics, blood loss, and other pathophysiological deficiencies leading to neurological complications. The rise in organ transplantation (heart. liver, lung, kidney) has led to significant increase in neurological problems observed by healthcare practitioners. Occasionally, these issues arise due to the unique characteristics of the transplant surgery, while in other cases, neurological impairment occurs because of subjecting an unwell patient to a lengthy and frequently challenging procedure.

### 2.1 Perioperative problems that may arise during every transplant operation

#### 2.1.1 Encephalopathy:

During the perioperative phase, individuals who have undergone transplantation may display a spectrum of behavioral alterations that can vary from a moderate state of disorientation or psychosis to a more severe encephalopathy characterized by obtundation or coma. During the first 48 hours following surgery, acute confusional episodes are typically associated with a widespread hypoxic-ischemic injury [34]. Additional potential factors encompass metabolic irregularities, impaired renal and hepatic function, the presence of multiple organ failure, and the occurrence of sepsis. When considering individuals with compromised hepatic and renal function, it is important to consider their reduced metabolism and excretion of anesthetics and other sedating drugs. The occurrence of altered mental status within a span of 2 to 5 days following surgery can potentially be attributed to acute care psychosis. This condition may be alleviated through the administration of neuroleptics or environmental reorientation [35].

Cyclosporine is associated with encephalopathy, a serious adverse reaction that affects around 5% of patients who are prescribed the medication [36]. The individuals in question may exhibit symptoms such as reduced cognitive awareness, cephalalgia, dysarthria, depressive symptoms, manic episodes, cortical blindness, visual hallucinations, and seizures [37]. Magnetic resonance imaging investigations can reveal extensive swelling and white matter abnormalities in the brain [36,37]. This condition is commonly observed in individuals with increased amounts of cyclosporine in their bloodstream. Nevertheless, it is important to note that other variables, including hypocholesterolemia, hypomagnesemia, high-dose steroids, hypertension, and uremia, may also play a role in its development [38,37].

#### 2.1.2 Seizure:

In patients undergoing transplants, seizures are a frequent occurrence, occurring in 6% to 36% of cases [39]. As usual, an underlying cause needs to be identified and a seizure should be treated as a sign of CNS dysfunction. Determining the type of seizure at onset (partial versus generalized) and its location is often beneficial. Among transplant recipients, medication toxicity (particularly cyclosporine, FK506, and OKT3), metabolic abnormalities, and hypoxic-ischemic damage are the most frequent causes of seizures [41]. Strokes and infections are less frequent sources of seizures. Another reason for seizures in this group is central nervous system infections, which typically develop weeks to months following organ transplantation.

#### 2.1.3 Neuropathies:

**Figure 1:**
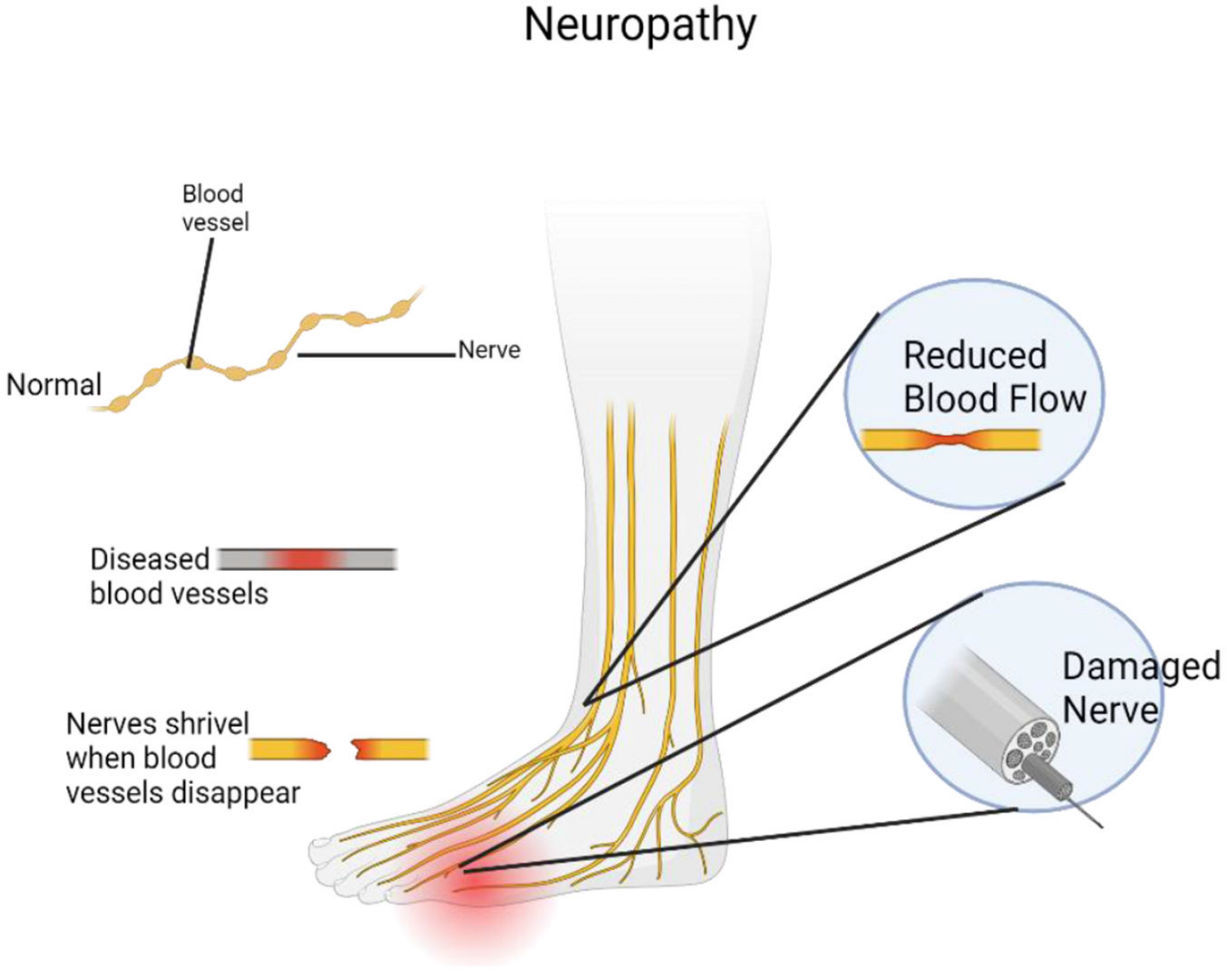
Peripheral neuropathy is a disorder caused from damage to nerve endings. Patients frequently report burning, itching, or shooting pain that often begins in the feet and gradually progresses to other areas. Peripheral neuropathy is a symptom of an underlying disease or process and needs testing to investigate the underlying cause.

Peripheral nerve damage can occur in organ transplantation, as well as in other extended surgical procedures (Figure 1). The prevalence varies between 5% among individuals who have undergone kidney transplantation and as high as 13% among patients who have undergone cardiac surgeries [42,35]. Peripheral nerve injuries can occur due to the mispositioning of patients who are pharmacologically paralyzed during surgery, stretching caused by prolonged retraction, or the creation of a local hematoma with compression of the nerve. The ulnar nerve, often located in the cubital tunnel, is the most frequently affected nerve under general anesthesia [43]. Injury at the elbow site may occur because of accentuated extension or flexion [43]. Every instance of transplantation is linked to a distinct pattern of peripheral nerve damage. For instance, renal transplant patients have been observed to exhibit femoral and lateral femoral cutaneous neuropathies [44], whereas heart transplant recipients have been found to experience lower brachial plexus stretch injuries [45]. Cold-induced phrenic nerve damage, which occurs when the heart is packed in ice during cardiac transplantation, is another recognized mechanism of nerve injury [35].

### 2.2 Perioperative complications unique to specific transplant procedures

#### 2.2.1 Kidney Transplantation:

The 1-year survival rate for transplant recipients is nearly 100%, with a graft survival rate ranging from 85% to 95% [46]. Notwithstanding these advancements, the incidence of neurologic problems associated with kidney transplants remain prevalent. Neurologic problems were observed in 30% of transplant recipients in a comprehensive study [47].

There are some neurological issues that are unique to the surgery, in addition to the potential risks associated with general anesthesia. The femoral and lateral femoral cutaneous nerves are frequently affected by compressive neuropathies following kidney donation [44]. Compressive femoral neuropathies, which typically result from the formation of local hematomas rather than retraction damage, are observed in a range of 1.5% to 8.4% among individuals who have had transplantation [48]. During the surgery, the lateral femoral cutaneous nerve is typically exposed and retracted. It is observed in 2.4% of patients in a specific study [44]. Renal transplantation can lead to an uncommon complication in patients who have abnormal blood flow to the lower part of the spinal cord. The caudal spinal cord in these patients receives blood supply via branches of the internal iliac artery, as opposed to the intercostal arteries. Spinal cord ischemia may occur when the iliac artery is utilized for blood flow to the allograft [49].

Cerebrovascular events are the prevailing neurologic sequelae following kidney transplantation, with a prevalence rate of 9.5% seen in a comprehensive retrospective analysis. Many of these incidents, however, took place at a period of more than 6 months after transplantation [50]. There is a notable correlation between the prevalence of cerebrovascular accidents and the underlying medical conditions that necessitate kidney transplantation, such as diabetes, hypertension, and lupus. Furthermore, it is common to find disruptions in the serum concentrations of cholesterol, triglycerides, and lipoproteins within this demographic [51]. Hematologic abnormalities and secondary polycythemia, which have been found in individuals who have undergone kidney transplantation, may also have a role in the occurrence of thromboembolic events [50].

#### 2.2.2 Liver Transplantation:

The scarcity of organs often results in an extended waiting period prior to the transplantation procedure. Consequently, individuals who undergo transplantation frequently exhibit severe illness and typically present with a certain level of hepatic encephalopathy prior to the surgical procedure [52]. Furthermore, this demographic has a higher susceptibility to hemorrhages in the central nervous system during the perioperative phase due to coagulopathies linked to liver failure.

The predominant perioperative neurologic complication observed in multiple clinical series following liver transplantation was encephalopathy, characterized by varying degrees of severity ranging from disorientation to coma [53-56]. Encephalopathy in this population can be attributed to various factors, such as metabolic abnormalities (including preexisting hepatic encephalopathy), medication toxicity (including cyclosporine and FK506), hypoxic ischemic brain damage, and infection. Autopsy studies conducted during the perioperative period indicate that metabolic and hypoxic-ischemic damage were the primary factors contributing to encephalopathy in liver transplant recipients [44,53,57].

The occurrence of central pontine myelinolysis is a rare neurological complication that is more prevalent among individuals who have undergone liver transplantation. central pontine myelinolysis has been regularly detected in 7% to 19% of liver transplant recipients in several neuropathologic studies [58-60]. The condition commonly leads to symmetric noninflammatory demyelination of the basis pontis, although neurons and axons are mostly spared [61]. Additionally, there have been reports of extrapontine myelinolysis [61].

The most prevalent peripheral nerve injury found after liver transplantation is injury to the lower brachial plexus, which can occur in up to 6% of patients [52,62]. The occurrence of the injury is thought to take place during the axillary dissection procedure that is necessary to gain access to the axillary vein for the purpose of endogenous bypass [52,62].

#### 2.2.3 Heart Transplantation:

While graft rejection and infection are significant contributors to death, it is worth noting that neurological problems might manifest in around 60% of patients, leading to substantial morbidity and subsequent impact on quality of life [63,64].

Cerebral infarction, encompassing both localized and diffuse ischemic-anoxic injury, is the prevailing neuropathological observation, with prevalence rates ranging from 16% to 43% in certain autopsy studies [65]. A retrospective analysis of clinical data showed a much lower incidence of neurological problems (5%) throughout the perioperative phase [66]. Cerebrovascular events can present as stroke with localized neurological impairments, disorientation, or severe encephalopathy accompanied by obtundation or coma. Global hypoxic-ischemic pathways are typically implicated in the occurrence of encephalopathy with psychosis during the immediate postoperative period. Encephalopathy may also manifest alongside metabolic derangements, multiple organ failure, and sepsis. Occasionally, seizures occurring immediately after surgery may be linked to focal embolic occurrences [67].

Cardiac transplantation is associated with a somewhat high incidence of peripheral nervous system damage, which are comparable to those observed in open heart surgery (13% of cases) [68]. The lower brachial plexus is frequently affected by injuries resulting from stretching during chest wall retraction or compression due to a hematoma. Individuals who have experienced lower brachial plexus injury commonly exhibit symptoms such as weakness and numbness in the distal arm, sometimes accompanied by a reduced triceps reflex. Tension can also cause recurrent laryngeal damage, which can lead to voice cord paralysis. Cold-induced injury resulting from the application of ice to the heart during transplantation has the potential to cause damage to the phrenic nerve [68]. Oftentimes, these individuals exhibit clinical symptoms such as diaphragmatic paralysis and challenges in transitioning away from mechanical ventilation or experiencing extended postoperative respiratory distress. Following the transplantation procedure, patients continue to face the potential for cerebrovascular events due to the presence of cardiac emboli, underlying atherosclerosis, and postoperative arrhythmias [69].

#### 2.2.4 Lung Transplantation:

There is limited information pertaining to the neurological problems that are linked to lung transplantation. Between October 1993 and October 1995, a total of 108 consecutive lung transplant procedures were conducted [70]. Out of the total number of transplant recipients, 21 individuals (19%) experienced 27 neurological problems. The most prevalent neurological consequence of transplantation was encephalopathy, which ranged from disorientation to coma, and affected 11 patients (10%) [70]. While metabolic reasons accounted for the bulk of cases (7.4%), medication toxicity also played a role in 2.8% of cases [70]. Four transplant recipients (3.7%) experienced seizures, which were attributed to metabolic abnormalities, cyclosporine toxicity, and stroke [70]. Cerebrovascular events were observed in a rather high frequency. Specifically, four patients (3.7%) experienced hypotensive watershed infarcts, four patients had embolic infarcts, and one patient had a hemorrhage in the vicinity of a watershed infarct. Two patients were found to have left atrial thrombi on transesophageal echocardiography during investigations into suspected sources of emboli. The pulmonary vein/left atrial anastomosis has the potential to serve as a significant reservoir of emboli in individuals who have undergone lung transplantation. In a study conducted by Stang et al. [71], a cerebral infarction was observed in a patient four weeks following a bilateral lung transplant. Transesophageal echocardiography revealed the presence of a thrombus in the left atrium at the location of the anastomosis. Furthermore, a study conducted on 21 individuals who underwent lung transplantation revealed the presence of pulmonary vein thrombosis in 5 patients (24%) during the immediate postoperative phase, as determined by transesophageal echocardiography [72].

### 2.3 Neurological complications after cardiac surgery

**Figure 2:**
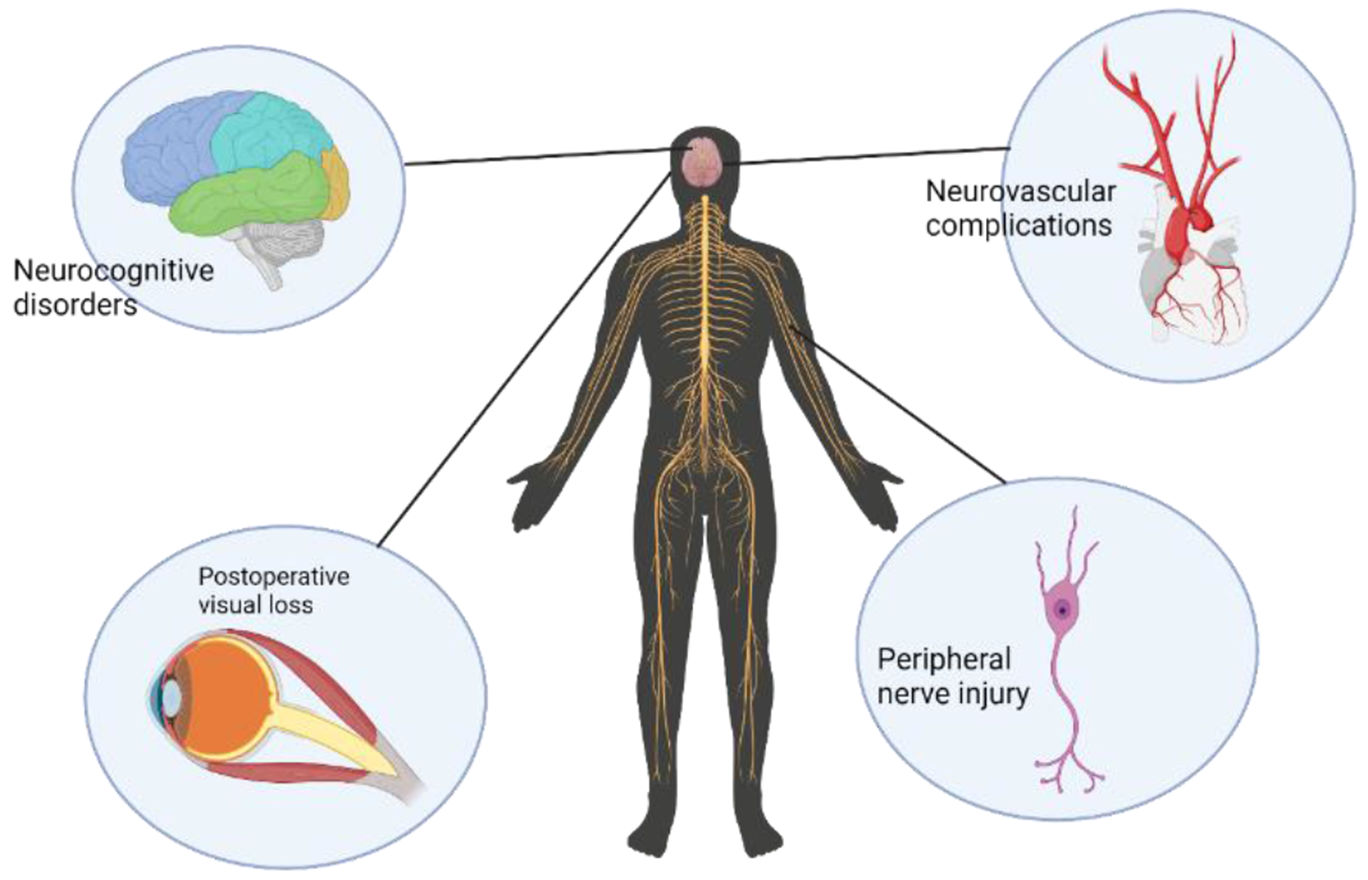
Potential Neurological complications in cardiac surgery.

Neurological problems rank as the second leading cause of morbidity and mortality after cardiac surgery, after heart failure. The occurrence of neurologic sequelae (Figure 2) substantially raises the probability of necessitating extended periods of care [73].

A significant complication following heart surgery is the occurrence of postoperative neurological impairment [74]. There are many different clinical symptoms, such as localized neurological abnormalities, altered states of consciousness, and cognitive-behavioral disorders [75]. Neurocognitive impairment is observed in a range of 15-66% of patients upon their release, and in a maximum of 40% of patients during a span of 5 years following the surgical procedure [76]. The prevalence of symptomatic stroke ranges from 1.2% to 6% among patients, with a larger occurrence observed in the geriatric population [77]. Multiple studies have assessed postoperative neurological problems and risk factors in patients undergoing cardiac surgery. However, the findings are inconsistent or insufficient mostly due to a limited number of interventions or variables. Several investigations have documented a correlation between extended cardiopulmonary bypass and the occurrence of postoperative stroke [77], but it has been subject to scrutiny by other researchers [78]. There is a lack of research assessing the occurrence of neurological problems after heart surgery in a significant number of individuals [76].

A research investigation was conducted to assess the prevalence of postoperative neurological issues among a cohort of 2,121 consecutive patients who underwent heart surgery. The findings revealed that 4.3% of the patients exhibited neurological abnormalities [79]. Out of the total of 2,121 patients, 37 individuals (1.7%) exhibited imaging evidence of stroke, whereas up to 71% displayed not-small ischemic strokes. The incidence of postoperative neurological problems has been found to be associated with higher rates of hospitalization and mortality.

#### 2.3.1 Bilateral Stenosis of Internal Carotid Artery:

With an odds ratio of 73.84, bilateral internal carotid artery (ICA) stenosis of any grade emerged as the most significant risk factor. Previous research has examined the correlation between stroke following cardiac surgery and carotid stenosis [80], which encompasses two primary mechanisms: artery-to-artery thrombosis (thrombosis from the proximal segment to the distal segment) or hypoperfusion (resulting in border zone infarcts - watershed infarction). Hirotani et al. (80) conducted a study using a sample of 476 patients who underwent coronary artery bypass grafting (CABG). The researchers identified carotid stenosis as the sole independent risk factor for postoperative stroke. As far as current research indicates, there is no established correlation between ICA stenosis, regardless of its severity, and neurologic impairment. Another study found that 69% of patients with neurological difficulties had bilateral ICA stenosis of any grade, even below 50%. In contrast, 85% of patients without neurological complications had normal ICAs [79].

### 2.4 Neurological Complications During Kidney transplant Anesthesia Procedures

Neurological problems are common in the context of renal illness and transplantation; in the posttransplant scenario, their incidence has been reported to range from 10% to 21%. Neurological side effects can happen soon after transplantation or they can take months or even years to manifest. Neurologic disorders can also result from renal disease; kidney transplantation may be able to alleviate or improve some of these disorders.

With over 17,000 kidney transplants performed each year and over 100,000 patients on the waiting list, kidney transplantation is the most common solid organ transplant in the United States [81]. Since kidney transplantation is more cost-effective over the course of a patient's life and, for most patients with end-stage renal disease, delivers better survival and quality of life than hemodialysis, this number has been rising over the past several years. However, difficulties necessitating hospitalization may arise during the first year following transplant, usually because of infection brought on by prolonged immunosuppression or immunosuppressive drugs. Between 30 and 60 percent of patients after a kidney transplant experience neurological problem, which are a prevalent problem [82]. Since neurological problems are linked to higher rates of morbidity and death, all patients receiving transplants should be evaluated for them.

#### 2.4.1 Ischemic stroke:

Ischemic strokes are prevalent among individuals who have undergone heart and kidney transplantation. Stroke has been observed in around 8% of individuals who have undergone renal transplantation. This occurrence might be attributed to factors such as hypertension, diabetes, and accelerated atherosclerosis, which can be acquired either during dialysis or following transplantation [82]. Ischemic strokes can be associated with various factors, including immunosuppressive treatment, surgical procedures such as transplantation, common surgical complications (such as bacterial infections, negative outcomes related to anesthesia, bleeding, etc.), the presence of bacterial endocarditis and vasoinvasive fungal central nervous system infections, hypercoagulable states, accelerated atherosclerosis, vasculitis, and cardiac arrhythmias after transplantation [82]. The causes of strokes in transplant recipients are primarily attributed to perioperative and infectious factors. However, in individuals who have survived for a medium to long period of time following transplantation, the occurrence of accelerated atherosclerosis can impact cerebrovascular circulation, hence elevating the risk of stroke. Strokes are observed in heart and liver allograft recipients at an early stage following transplantation, while they tend to manifest at a later stage following kidney transplantation. Age over 40 and diabetes were identified as significant risk factors for cerebrovascular illness in kidney transplant recipients [85]. Atherosclerosis is often associated with ischemic strokes following kidney donation [86]. A notable decrease in the occurrence of strokes has been documented in individuals with diabetes who have concurrent kidney and pancreas transplantation [84].

The incidence of stroke is higher following kidney transplantation, whereas liver transplantation is associated with encephalopathy, seizures, and central pontine myelinolysis. Stroke is the predominant neurological complication in kidney transplant patients, but cerebral infarction and hemorrhage are more commonly observed after heart transplantation. Post-transplant polycythemia and hypercoagulability are commonly observed in kidney allograft recipients, with a prevalence rate of 17% among patients. These conditions have been associated with an elevated risk of stroke [87]. Kidney allograft recipients transplanted for polycystic kidney disease have a 10-fold higher chance of experiencing aneurismal subarachnoid hemorrhage [88]. The occurrence of diabetic nephropathy as a contributing factor to pre-transplant kidney failure has been found to elevate the likelihood of post-transplant cerebral hemorrhage [88]. It is reasonable to expect that a similar outcome may arise from the use of immunosuppressants in individuals with diabetes. Calcineurin inhibitors and steroids are primarily responsible for causing post-transplant diabetes, with tacrolimus being associated with a higher likelihood of this problem. A retrospective review of kidney transplant recipients revealed a higher incidence of post-transplantation diabetes mellitus in patients who received a cadaver organ compared to those who received a living donor organ. Additionally, this condition was more commonly observed in patients who received tacrolimus therapy vs cyclosporine therapy [89]. However, it has been observed that the use of mTOR inhibitors is linked to a higher likelihood of developing new-onset diabetes after transplantation (NODAT) [90].

#### 2.4.2 Encephalopathy:

**Figure 3:**
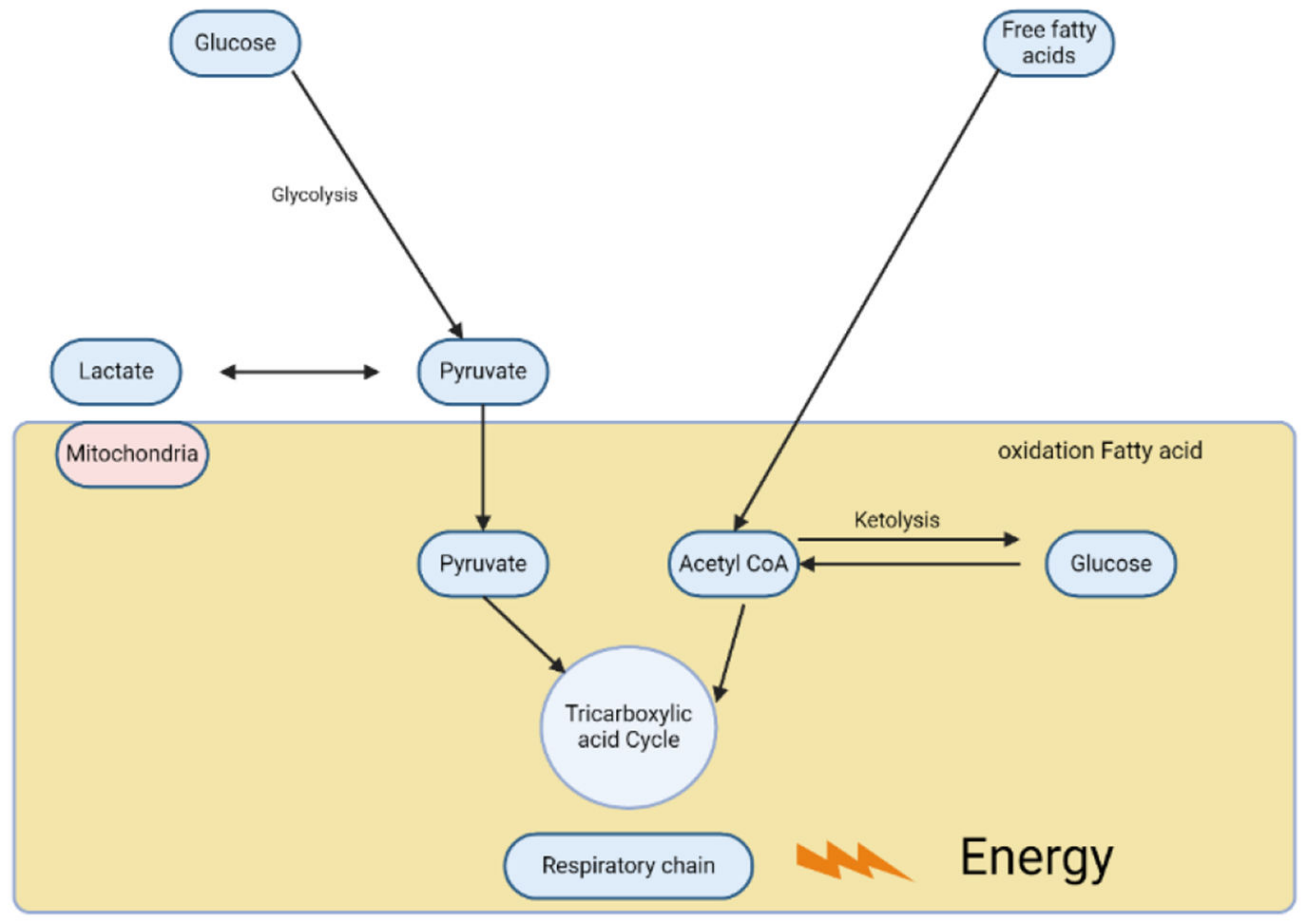
Energy failure model of metabolic encephalopathy. Hypoglycemia and any enzyme defect in mitochondrial energy production may result in metabolic encephalopathy. The pathophysiology of metabolic encephalopathy by fatty acid oxidation defect is multifactorial.

Metabolic encephalopathy frequently occurs in transplant recipients, with its causes being as diverse as in non-transplant patients (Figure 3). These causes often involve alterations in electrolyte levels and glucose levels. Individuals who have pre-existing diabetes exhibit sensitivity to steroid treatment, and the administration of Tac may potentially trigger the onset of diabetic ketoacidosis. Discontinuation of steroids abruptly and unintentionally might also lead to a disruption in consciousness. Individuals who experience a severe systemic infection without central nervous system involvement commonly develop encephalopathy associated with sepsis [91]. Hepatic and uremic encephalopathy have been found to be related with delayed graft function in orthotopic liver and kidney transplantation, respectively [91].

#### 2.4.3 Acute neuropathy:

Acute neuropathy can also result during surgery, although peripheral neuropathy following kidney transplantation can also be brought on by drugs used for immunosuppression and antimicrobial prophylaxis. With a frequency of between 2% and 4%, acute femoral neuropathy is a rather uncommon consequence (92). It is thought that the pathophysiology consists of direct compression and ischemia of the nerve, which may be brought on by the steal phenomenon after the graft renal artery and the iliac artery have anastomose.

#### 2.4.4 Stroke:

Cerebrovascular illness is a commonly observed condition among individuals who have received kidney transplantation, exhibiting a stroke or transient ischemic attack rate of 5% within the initial year and 9.4% within the subsequent year (93). A significant portion of the occurrence can be attributed to the frequent presence of comorbidities among individuals who have undergone renal transplantation. Factors such as diabetes, advanced age, post-transplant kidney function, and prior stroke are all recognized as risk factors for ischemic stroke following transplantation. Conversely, diabetes, precipitated kidney disease, and hypertension have been found to be associated with hemorrhagic stroke (94). Post-transplant polycythemia and hypercoagulability can be observed in renal patients, hence elevating the susceptibility to stroke.

### 2.5 Neurological complications during open radical prostatectomy, cystectomy

#### Robot-assisted laparoscopic prostatectomy: Neuropathic pain consequences resulting from lesions in the endopelvic nerve:

The most often employed approach for surgical intervention in individuals with localized prostate cancer is robot-assisted laparoscopic prostatectomy (RALP), which is the most often used surgical approach for treating patients with localized prostate cancer. The RALP approach provides several significant benefits compared to traditional retropubic radical prostatectomy. These advantages include decreased blood loss, reduced postoperative pain, faster recovery, shorter hospital stays, and improved outcomes in terms of postoperative urinary continence and erectile function [95].

Neurological damage linked with RALP can still occur, even when performed by surgeons with extensive experience. Patient malposition on the surgical table or endopelvic injury after surgery can lead to neurological problems. Currently, there is a growing recognition of the possibility of nerve damage caused by robotic-assisted laparoscopic surgery [96] and the need to prevent them. Numerous inquiries have been conducted to examine the quantitative aspects of this subject matter. As an illustration, a retrospective examination of 377 RALP procedures revealed that the incidence of postoperative neuropathies was 1.3% [97]. In recent studies, Wen et al. (1998) documented a peripheral nerve injury incidence rate of 0.16%, whereas Gezginci et al. (99) observed neuromuscular problems in around 5% of the patient population.

RALP surgery can result in several types of surgical damage to the pelvic nerves. Various processes are involved in the pathophysiology of these damages, including compression (e.g., caused by hematoma or pelvic lymphoceles), transection, incision, traction, thermal traumas, and trapping with clips. The combination of these mechanisms is most observed during the Santorini plexus stitch, Rocco stitch, or PLND. Most of these pathways have the potential to cause significant damage to Schwann cells and the loss of myelin, resulting in long-lasting nerve damage [100].

### 2.6 Neurological complications during Major Oncologic Head and Neck Surgery

#### Neurovascular Complications after Neck Dissection:

Due to the prevalence of oral malignancies, neck dissection is a frequently conducted technique within the field of head and neck oncology. This surgical procedure is especially susceptible to a range of neurovascular problems because of the complex architecture of the neck.

With the advancement of neck dissection surgical procedures, there has been a corresponding increase in the kind and occurrence of problems. Neck dissections are susceptible to a range of neurovascular problems because to their complex anatomical structure and the presence of numerous nerves, arteries, and lymphatic channels. Neck dissection frequently leads to shoulder complaints and functional impairment, which are often caused by injury to the spinal accessory nerve either from dissection or direct impact. Despite efforts to preserve the spinal accessory nerve during neck dissection, a considerable number of patients nevertheless experience varying degrees of shoulder impairment. In addition to adhesive capsulitis and myofascial pain in the upper trapezius, levator scapulae, and rhomboid muscles, these symptoms can be related to spinal accessory neuropraxia and neurotmesis [101]. The greater auricular nerve (GAN) and marginal mandibular nerve (MMN) are also commonly injured nerves. However, the incidence of phrenic nerve injury is rather modest [102].

Neck dissection, primarily performed on the left side, poses a potential risk of chylous fistula, which can occur in approximately 3-5% of instances. Previous research has indicated that the incidence of internal jugular vein (IJV) thrombosis following neck dissection varies between 0% and 29.6% [103].

Both marginal mandibular and greater auricular nerve praxia following neck dissection are more prevalent than commonly perceived, yet frequently overlooked. This is because during the post-operative phase, both the surgeon and patient are preoccupied with other significant post-operative matters associated with complex surgical procedures, such as feeding, pain management, and wound care.

Phrenic nerve paralysis is a nerve complication that can occur because of neck dissection. It is characterized by the elevation of the hemidiaphragm on the same side, with or without a shift in the mediastinal plane on a chest radiograph. This condition has the potential to cause complications in the lungs throughout the post-operative period. The fundamental approach to injury prevention involves the preservation of the fascial layer that covers the nerve and anterior scalene muscle. Unilateral phrenic nerve paralysis was seen in 14 cases (8%) out of 176 consecutive neck dissections in a retrospective analysis. Severe symptoms were not observed in any of the patients who experienced post-operative phrenic nerve paralysis [104].

Chylous leaking is a rare complication that can occur following neck dissection. Various therapeutic methods have been identified for this condition. The incidence of chylous fistula after radical neck dissection is typically between 1% and 2.5%, with the majority (75-92%) occurring on the left side [105]. Teymoortash et al. [106] conducted a study where 2 out of 98 patients who underwent selective neck dissection experienced chylous leak after the surgery. These cases were treated with conservative measures [106].

Post-operative thrombosis is a potential complication of neck dissection, in addition to intra-operative damage or closure of the internal jugular vein. The study conducted by Quraishi et al. (year) documented an initial incidence of IJV thrombosis at 25%, which subsequently dropped to 6% during the long-term follow-up period [107]. Previous research has documented a range of incidence rates for IJV thrombosis, varying from 0% to 29.6%. The studies conducted by Harada et al. [103] and Teymoortash et al. [106] have documented a patency rate of 100% following neck dissection.

In the last century, neck dissection has emerged as the prevailing procedure in the field of head and neck oncology, whether conducted independently or as an essential component of extensive resection and reconstruction. Despite its regularity, it is important not to underestimate the related morbidities and make every effort to reduce them by becoming familiar with the intricate anatomy of the region.

### 2.7 Neurological complications during Spine deformity surgery

Given the aging population, increased life expectancy, and evidence of improved health-related quality of life following surgical intervention, there has been a rise in the number of adult spinal deformity (ASD) procedures. Still, there is a significant likelihood of medical and surgical consequences. Patients may experience particularly severe neurological effects, such as spinal cord injury and motor impairments. Anterior, lateral, or posterior surgical approaches, the use of osteotomies, thoracic hyperkyphosis, the location of the spine, patient characteristics, and the status of revision surgery are some of the independent variables that may have an impact on the chance of neurological issues.

Any illness that causes aberrant spinal alignment in the axial, coronal, and sagittal planes is classified as an ASD [108]. The prevalence of ASD is expected to rise over the next few decades because of an aging population, longer life expectancies, and increased awareness, with over 60% of adults over 60 having the disorder [109,110].

Changes in anatomy, the need for osteotomies due to fusion masses, scar tissue, and the presence of neurological abnormalities prior to surgery, in comparison to primary surgery patients, may make revision surgery a risk factor for neurological problems [111]. The odds ratio (OR) for nervous system injury (1.10-1.60) and accidental vessel or nerve puncture (1.44 [1.29-1.61]) were higher in revision surgery patients (72 vs. 47%, p = 0.0001) and those who had the procedure performed nationwide [111]. A analysis of 108,419 spine procedures conducted in another study [112] found that the rate of neurological problems following revision surgery was 41% greater than that of first surgery, providing further evidence of this elevated risk of difficulties. Notwithstanding the fact that the revision group required a considerably higher number of three-column osteotomies (3-COs) than the main cohort, Hassanzadeh et al. [113] did not detect a statistically significant increased rate of severe complications in their comparison of 167 primary and revision ASD procedures.

Following surgery for adult cervical deformity, a prospective multicenter center with 78 patients found a major complication rate of 24%, with C5 motor palsies accounting for 6.4% of cases [114]. The study conducted by He and colleagues (115) examined 316 patients who underwent posterior cervical fusions for congenital and mixed etiologies of abnormalities. Spontaneous spinal cord injury accounted for 1.3% of cases during surgery, with reperfusion following decompression being the most common cause. 1 percent of cases had nerve root injury, which was traced to incorrect lateral mass screw placement and verified by electromyography monitoring. A further case of spinal cord injury following intubation [116] describes cervical hyperextension.

When anesthetic methods are used to treat the cervical spine, vertebral artery injury (VAI) is a potentially harmful outcome that can have a neurological impact. Lall et al. [117] examined the complications of 2,274 craniometrical fusion procedures and discovered a 1.3-2.1% VAI rate when C1-2 trans articular screws were inserted. Since the injury in these cases was more common, the authors stressed the importance of identifying a high-riding vertebral artery. Identifying additional anomalies such as a convoluted vertebral artery, a high entrance level into the transverse foramen, and a persisting first intersegmental artery is also crucial. According to another study [118], 3.7% of individuals with unilateral VAIs experienced neurological sequelae. After anterior cervical surgery, nerve injuries, including those to the recurrent laryngeal nerve (1.3–13.3 %) [119], superior laryngeal nerve (1%), hypoglossal nerve (<1%) [120], and cervical sympathetic chain (0.2-4%), have been properly documented [121].

### 2.8 Neurological complications during Aortic Surgery

Aortic surgery can obstruct blood flow from the aorta to the nervous system, which can lead to ischemic neurologic symptoms. While aortic procedures are a relatively new area of surgical advancement, the neurologic consequences of aortic blockage have long been studied and discussed. The occurrence was initially documented in 1667 when a rabbit's hindquarter was paralyzed following obstruction of the abdominal aorta, as reported by Stenonis and Swammerdam [122]. The Stenonis experiment is the name given to this observation. The cause of this paralysis remained unknown and was first thought to be either peripheral nerve or medulla ischemia [122]. However, Schiffer's research in 1869 disproved both theories. He found that the difference between suprarenal and infrarenal degrees of aortic occlusion in the dog determined whether the occlusion was central or peripheral. In 1899, Hoche elucidated that the dog's spinal circulation differed from that of the rabbit, making the Stenonis experiment unfeasible in that species. Neurological consequences following aortal ligation in humans were a topic of intense debate and discussion in the past. Many authors in the earlier literature on the pathogenesis of neurologic injury following saddle embolism acknowledged an ischemic origin in the peripheral nerve, but others were swayed by the rabbit experiment results and supported a spinal origin in such circumstances.

### 2.9 Neurological complications caused by ischemia of peripheral nerves

**Figure 4:**
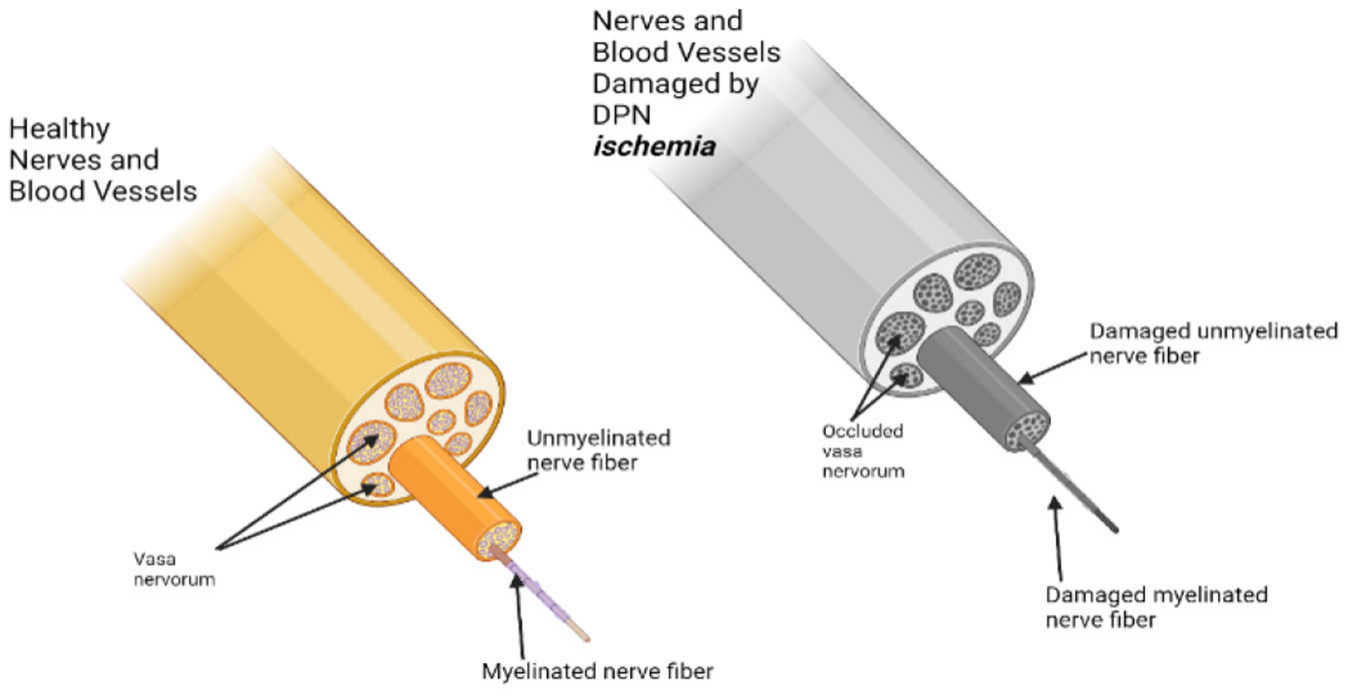
Schematic diagram showing the effect of ischemia due to diabetic peripheral neuropathy (DPN) leading to occluded vasa vasorum, damaged unmyelinated and myelinated nerve fibers.

The extension of the disease beyond this field may need prolonged closure of the abdominal aorta during abdominal aortic surgery. Saddle embolism is another possible cause. This is a risk associated with any aortic procedure, but it is more so after aneurysm surgery, as surgery is one of the etiologic causes of saddle embolism. Dissecting aneurysms that have been documented as the consequence of aortic surgery may also cause prolonged blockage of the abdominal aorta [122]. The neurological complications are exaggerated with the co-morbidity of diabetes (Figure 4).

### 2.10 Neurological complications after thoracic endovascular aortic repair

**Figure 5:**
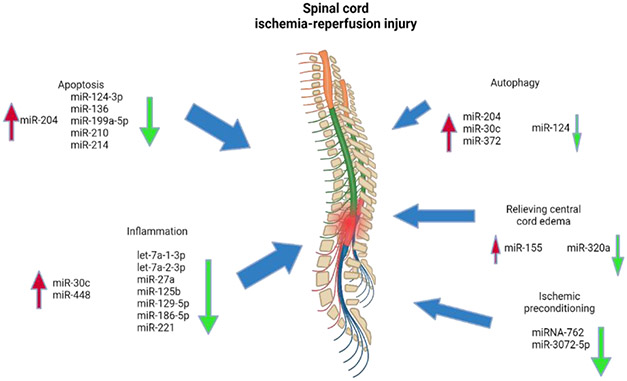
Several microRNAs (miRNAs) play a role in the pathophysiology of spinal cord ischemia-reperfusion injury to induce inflammation and damage due to apoptosis and autophagy of the cells.

Spinal cord ischemia (SCI) and ischemic stroke rank among the most serious side effects of TEVAR. Spent grafts that cover a large portion of the aorta wall, blocking the arteries providing blood to the spinal cord, are the cause of lower limb paresis and spinal cord ischemia. Despite being thought to be lower than in open surgery, the risk of stroke and SCI can nevertheless reach 2.5-5% [123]. Neurological problems are linked to long-term disability and handicap, which results in substantial costs. They can be deadly in up to 30% of cases. Several microRNAs (miRNAs) have been implicated in the underlying pathophysiology of spinal cord ischemia-reperfusion injury. Various cellular events and rise and fall of specific miRNAs induce inflammation and damage due to apoptosis and autophagy of the cells (Figure 5).

## 3 Conclusion

Even with the technological advancements in organ transplantation, neurologic problems continue to be a major cause of morbidity and death. All transplant types share many of the same problems, such as encephalopathy, seizures, and damage to peripheral nerves. Additional problems are specific to each transplant operation. The process of kidney transplantation is generally benign and does not carry a high risk of perioperative morbidity. Significant neurological side effects from liver transplantation include central pontine myelinolysis and hepatic encephalopathy. Both hypoxic-ischemic damage and cerebral infarction are frequently linked to heart and lung transplants, often resulting from a cardiac embolus. The annual number of transplants performed is rising, thus it is critical that the neurologists understand the potential issues associated with organ transplantation. When it comes to identifying and treating neurological side effects of transplantation, neurologist expertise is crucial.

## References

[1] Blazer DG, Nieuwenhuizen AO. Evidence for the diagnostic criteria of delirium. Current Opinion in Psychiatry 25 (2012): 239–243. 22449764 10.1097/YCO.0b013e3283523ce8

[2] Jabbar F, Leonard M, Meehan K, Neuropsychiatric and cognitive profile of patients with DSM-IV delirium referred to an old age psychiatry consultation-liaison service. International Psychogeriatrics 23 (2011): 1167–1174. 21251353 10.1017/S1041610210002383

[3] Neufeld KJ, Leoutsakos JMS, Sieber FE, Outcomes of Early Delirium Diagnosis After General Anesthesia in the Elderly. Anesthesia & Analgesia 117 (2013): 471–478. 23757476 10.1213/ANE.0b013e3182973650 PMC4017627

[4] Vlajkovic GP, Sindjelic RP. Emergence Delirium in Children: Many Questions, Few Answers. Anesthesia & Analgesia 104 (2007): 84–91. 17179249 10.1213/01.ane.0000250914.91881.a8

[5] Saczynski JS, Marcantonio ER, Quach L, Cognitive Trajectories After Postoperative Delirium. Survey of Anesthesiology 56 (2012): 286–287. 10.1056/NEJMoa1112923 PMC3433229 22762316

[6] Meagher DJ, Leonard M, Donnelly S, (2012, March). A longitudinal study of motor subtypes in delirium: Frequency and stability during episodes. Journal of Psychosomatic Research 72 (2012): 236–241. 22325705 10.1016/j.jpsychores.2011.11.013

[7] Cerejeira J, Nogueira V, Luís P. The Cholinergic System and Inflammation: Common Pathways in Delirium Pathophysiology. Journal of the American Geriatrics Society 60 (2012): 669–675. 22316182 10.1111/j.1532-5415.2011.03883.x

[8] Hughes C, Morandi A, Girard T, Association between Endothelial Dysfunction and Acute Brain Dysfunction during Critical Illness. Anesthesiology 118 (2013): 631–639. 23263016 10.1097/ALN.0b013e31827bd193 PMC3580006

[9] Lawlor PG, Gagnon B, Mancini IL, Occurrence, Causes, and Outcome of Delirium in Patients With Advanced Cancer. Archives of Internal Medicine 160 (2000): 786. 10737278 10.1001/archinte.160.6.786

[10] Clegg A, Young JB. Which medications to avoid in people at risk of delirium: a systematic review. Age And Ageing 40 (2010): 23–29. 21068014 10.1093/ageing/afq140

[11] Sieber FE, Zakriya KJ, Gottschalk A, Sedation Depth During Spinal Anesthesia and the Development of Postoperative Delirium in Elderly Patients Undergoing Hip Fracture Repair. Mayo Clinic Proceedings 85 (2010): 18–26. 20042557 10.4065/mcp.2009.0469 PMC2800291

[12] Mason SE, Noel-Storr A, Ritchie CW. The Impact of General and Regional Anesthesia on the Incidence of Post-Operative Cognitive Dysfunction and Post-Operative Delirium: A Systematic Review with Meta-Analysis. Journal of Alzheimer’s Disease 22 (2010): S67–S79. 10.3233/JAD-2010-101086 20858956

[13] McDonagh D, Mathew J, White W, Cognitive Function after Major Noncardiac Surgery, Apolipoprotein E4 Genotype, and Biomarkers of Brain Injury. Anesthesiology 112 (2010): 852–859. 20216394 10.1097/ALN.0b013e3181d31fd7 PMC2933423

[14] Evered L, Scott DA, Silbert B, Postoperative Cognitive Dysfunction Is Independent of Type of Surgery and Anesthetic. Survey of Anesthesiology 56 (2012): 19. 10.1213/ANE.0b013e318215217e 21474666

[15] Monk T, Weldon B, Garvan C, Predictors of Cognitive Dysfunction after Major Noncardiac Surgery. Anesthesiology 108 (2008): 18–30. 18156878 10.1097/01.anes.0000296071.19434.1e

[16] Steinmetz J, Christensen K, Lund T, Long-term Consequences of Postoperative Cognitive Dysfunction. Anesthesiology 110 (3): 548–555. 19225398 10.1097/ALN.0b013e318195b569

[17] Abildstrom H, Rasmussen LS, Rentowl P, Cognitive dysfunction 1-2 years after non-cardiac surgery in the elderly. Acta Anaesthesiologica Scandinavica 44 (2000): 1246–1251. 11065205 10.1034/j.1399-6576.2000.441010.x

[18] Selnes OA, Gottesman RF, Grega MA, Cognitive and Neurologic Outcomes After Coronary-Artery Bypass Surgery. Survey of Anesthesiology 56 (2012): 212–213. 10.1056/NEJMra1100109 22256807

[19] Selnes OA, Grega MA, Bailey MM, Cognition 6 years after surgical or medical therapy for coronary artery disease. Annals of Neurology 63 (2008): 581–590. 18481292 10.1002/ana.21382

[20] Sauër AMC, Nathoe HM, Hendrikse J, Cognitive Outcomes 7.5 Years After Angioplasty Compared With Off-Pump Coronary Bypass Surgery. The Annals of Thoracic Surgery 96 (2013): 1294–1300. 23866798 10.1016/j.athoracsur.2013.05.001

[21] Avidan MS, Evers AS. Persistent Postoperative Cognitive Decline? Anesthesiology 124 (2016): A23–A23. 26785428 10.1097/ALN.0000000000000958 PMC5839806

[22] Terrando N, Monaco C, Ma D, Tumor necrosis factor-α triggers a cytokine cascade yielding postoperative cognitive decline. Proceedings of the National Academy of Sciences 107 (2010): 20518–20522. 10.1073/pnas.1014557107 PMC2996666 21041647

[23] Cormack F, Shipolini A, Awad WI, A meta-analysis of cognitive outcome following coronary artery bypass graft surgery. Neuroscience & Biobehavioral Reviews 36 (2012): 2118–2129. 22732162 10.1016/j.neubiorev.2012.06.002

[24] Bucerius J, Gummert JF, Borger MA, Stroke after cardiac surgery: a risk factor analysis of 16,184 consecutive adult patients. The Annals of Thoracic Surgery 75 (2003): 472–478. 12607656 10.1016/s0003-4975(02)04370-9

[25] Moneta G General anaesthesia versus local anaesthesia for carotid surgery (GALA): a multicentre, randomised controlled trial. Yearbook of Vascular Surgery 11 (2009): 224–225.

[26] Mashour GA, Shanks AM, Kheterpal S. Perioperative Stroke and Associated Mortality after Noncardiac, Nonneurologic Surgery. Anesthesiology 114 (2011): 1289–1296. 21478735 10.1097/ALN.0b013e318216e7f4

[27] Sharifpour M, Moore LE, Shanks AM, Incidence, Predictors, and Outcomes of Perioperative Stroke in Noncarotid Major Vascular Surgery. Anesthesia & Analgesia 116 (2013): 424–434. 23115255 10.1213/ANE.0b013e31826a1a32

[28] Mrkobrada M, Hill MD, Chan MT, Abstract TMP9: The Neurovision Pilot Study: Non-cardiac Surgery Carries A Significant Risk Of Acute Covert Stroke. Stroke 44 (2013): 23.

[29] Lintott P, Hafez HM, Stansby G. Spinal cord complications of thoracoabdominal aneurysm surgery. British Journal of Surgery 85 (1998): 5–15. 9462373 10.1046/j.1365-2168.1998.00658.x

[30] Acher CW, Wynn MM, Mell MW, A Quantitative Assessment of the Impact of Intercostal Artery Reimplantation on Paralysis Risk in Thoracoabdominal Aortic Aneurysm Repair. Annals of Surgery 248 (2008): 529–540. 18936565 10.1097/SLA.0b013e318187a792

[31] Conrad MF, Ye JY, Chung TK, Spinal cord complications after thoracic aortic surgery: Long-term survival and functional status varies with deficit severity. Journal of Vascular Surgery 48 (2008): 47–53. 18486422 10.1016/j.jvs.2008.02.047

[32] Shen Y, Drum M, Roth S. The Prevalence of Perioperative Visual Loss in the United States: A 10-Year Study from 1996 to 2005 of Spinal, Orthopedic, Cardiac, and General Surgery. Anesthesia & Analgesia 109 (2009): 1534–1545. 19713263 10.1213/ane.0b013e3181b0500b

[33] Patil CG, Lad EM, Lad SP, Visual Loss After Spine Surgery. Spine 33 (2008): 1491–1496. 18520945 10.1097/BRS.0b013e318175d1bf

[34] Hotson JR, Enzmann DR. Neurologic Complications of Cardiac Transplantation. Neurologic Clinics 6 (1988): 349–365. 3047545

[35] Sila CA. Spectrum of neurologic events following cardiac transplantation. Stroke 20 (1989): 1586–1589. 2554543 10.1161/01.str.20.11.1586

[36] Neurologic complications in allogeneic bone marrow transplant patients receiving cyclosporin (1991). 1768975

[37] Walker RW, Brochstein JA. Neurologic Complications of Immunosuppressive Agents. Neurologic Clinics 6 (1988): 261–278. 3138519

[38] Kahan BD. Immunosuppressive therapy with cyclosporine for cardiac transplantation. Circulation 75 (1987): 40–56. 3539397 10.1161/01.cir.75.1.40

[39] Estol CJ, Pessin MS, Martinez AJ. Cerebrovascular complications after orthotopic liver transplantation. Neurology 41 (1991): 815–815. 2046922 10.1212/wnl.41.6.815

[40] Gilmore RL. Seizures and Antiepileptic Drug Use in Transplant Patients. Neurologic Clinics 6 (1988): 279–296. 3047542

[41] Pruitt A Neurological complications after solid organ transplantation. Journal of the Neurological Sciences 357 (2015): e483.

[42] Huang ST, Yu TM, Chuang YW, The Risk of Stroke in Kidney Transplant Recipients with End-Stage Kidney Disease. International Journal of Environmental Research and Public Health 16 (2019): 326. 30682846 10.3390/ijerph16030326 PMC6388105

[43] Borgeat A, Ekatodramis G. Nerve Injury Associated with Regional Anesthesia. Current Topics in Medicinal Chemistry 1 (2001): 199–203. 11895136 10.2174/1568026013395272

[44] Van Veer H, Coosemans W, Pirenne J, Acute Femoral Neuropathy: A Rare Complication After Renal Transplantation. Transplantation Proceedings 42 (2010): 4384–4388. 21168704 10.1016/j.transproceed.2010.07.013

[45] Neurological Complications of On-Pump versus Off-Pump Coronary Artery Bypass Graft Surgery. International Journal of Science and Research 6 (2017): 2174–2177.

[46] Schrem H Risk Factors for Short- and Long-Term Mortality in Liver Transplant Recipients with MELD Score ≥30. Annals of Transplantation 20 (2015): 59–69. 25630462 10.12659/AOT.892322

[47] Cheng CY, Wang HY, Liou WS, Hazards of Stroke in Renal Transplant Recipients and Patients With End-Stage Renal Disease. Transplantation Proceedings 51 (2019): 1402–1405. 31076151 10.1016/j.transproceed.2019.01.138

[48] Chang I, Kim T. Incidence of femoral neuropathy after kidney transplantation. Transplantation 86 (2008): 760.

[49] Jablecki CK, Aguilo JJ, Piepgras DG, Paraparesis after renal transplantation. Annals of Neurology 2 (1977): 154–155.

[50] Prevalence and Risk Factors of Noncontrolled and Resistant Arterial Hypertension in Renal Transplant Recipients. Transplantation 100 (2016): e54. 10.1097/TP.0000000000001424 27548848

[51] Malviya P, Maitra S. Renal transplantation in polycythemia vera- A rare case report from India. Indian Journal of Transplantation 14 (2020): 333.

[52] Patchell RA. Neurological complications of organ transplantation. Annals of Neurology 36 (1994) 688–703. 7979215 10.1002/ana.410360503

[53] Moreno E, Gómez SR, Gonzalez I, Neurologic complications in liver transplantation. Acta Neurologica Scandinavica 87 (2009): 25–31. 10.1111/j.1600-0404.1993.tb04070.x 8380946

[54] Pujol A, Graus F, Rimola A, Predictive factors of in-hospital CNS complications following liver transplantation. Neurology 44 (1994): 1226–1236. 8035920 10.1212/wnl.44.7.1226

[55] Erol I, Alehan F, Ozcay F, Neurological complications of liver transplantation in pediatric patients: A single center experience. Pediatric Transplantation 11 (2006): 152–159. 10.1111/j.1399-3046.2006.00620.x 17300494

[56] Munday G, Eng M, Klink F, An Uncommon Neurologic Complication Following Orthotopic Liver Transplantation. American Journal of Transplantation 13 (2013): 2496–2498. 24034711 10.1111/ajt.12346

[57] Živković SA. Neurologic complications after liver transplantation. World Journal of Hepatology 5 (2013): 409. 24023979 10.4254/wjh.v5.i8.409 PMC3767839

[58] Yu J. Possible causes of central pontine myelinolysis after liver transplantation. World Journal of Gastroenterology 10 (2004): 25–40. 10.3748/wjg.v10.i17.2540 PMC4572157 15300900

[59] Sato K, Sekiguchi S, Akamatsu Y, Liver laceration associated with severe seizures after living donor liver transplantation. Liver Transplantation 12 (2005): 152–155. 10.1002/lt.20629 16382468

[60] Soliman AR, Mamoun H, Zayed B. The use of cyclosporin a and mycophenolate mofetil 48 hours before renal transplantation enables the use of a low dose cyclosporin and better graft function. Transplantation Journal 90 (2010): 632. 10.1016/j.transproceed.2010.09.065 21168619

[61] Lee EM, Kang JK, Yun SC, Risk Factors for Central Pontine and Extrapontine Myelinolysis following Orthotopic Liver Transplantation. European Neurology 62 (2009): 362–368. 19797900 10.1159/000242426

[62] Dulitz MG, De Wolf AM, Wong H, Compression of the brachial plexus during right lobe liver donation as a cause of brachial plexus injury: A case report. Liver Transplantation 11 (2005): 233–235. 15666376 10.1002/lt.20343

[63] Biller J, Hocker S, Morales-Vidal S. Neurologic Complications of Cardiac Surgery and Interventional Cardiac Procedures. Hospital Practice 38 (2010) 83–89. 21068531 10.3810/hp.2010.11.344

[64] Power A, Murray J, Dykes J, Neurologic Events Following Pediatric Heart Transplantation. The Journal of Heart and Lung Transplantation 40 (2021): S198–S199.

[65] Montero CG, Martinez AJ. Neuropathology of heart transplantation. Neurology 36 (1986): 1149–1149. 3528916 10.1212/wnl.36.9.1149

[66] Mainali S Neurologic Complications of Cardiac and Pulmonary Disease. CONTINUUM: Lifelong Learning in Neurology 29 (2023): 684–707. 37341327 10.1212/CON.0000000000001284

[67] The significance of neurological complications in heart transplant recipients. Transplantation 82 (2006): 563–564.

[68] Perona. (2013). Drug Interactions With Cyclosporine-Rifampicin-Clarithromycin in a Kidney Transplant Recipient With Mycobacterium Malmoense. Journal of Medical Cases.

[69] Gill W Cerebrovascular complications of cardiac transplantation. Seminars in Cerebrovascular Diseases and Stroke 3 (2003): 219–224.

[70] Lee JM, Raps EC. Neurologic complications of transplantation. Neurologic Clinics 16 (1998): 21–33. 9421539 10.1016/s0733-8619(05)70365-0

[71] Stang MR, Hinderliter AL, Gott KK, Atrial anastomotic thrombus causes neurologic deficits in a lung transplant recipient. Transplantation 62 (1996): 693–695. 8830840 10.1097/00007890-199609150-00028

[72] Hillier S, Roper D, Harten M, Echocardiographic Assessment of Pulmonary Vein Velocities Following Lung Transplantation. Heart, Lung and Circulation 22 (2013): S176.

[73] Floyd TF, Cheung A, Stecker MM. Postoperative Neurologic Assessment and Management of the Cardiac Surgical Patient. Seminars in Thoracic and Cardiovascular Surgery 12 (2000): 337–348. 11154729 10.1053/stcs.2000.20040

[74] McKhann GM, Grega MA, Borowicz LM, Stroke and Encephalopathy After Cardiac Surgery. Stroke 37 (2006): 562–571. 16373636 10.1161/01.STR.0000199032.78782.6c

[75] Newman MF, Grocott HP, Mathew JP, Report of the Substudy Assessing the Impact of Neurocognitive Function on Quality of Life 5 Years After Cardiac Surgery. Stroke 32 (2001): 2874–2881. 11739990 10.1161/hs1201.099803

[76] Bucerius J, Gummert JF, Borger MA, Stroke after cardiac surgery: a risk factor analysis of 16,184 consecutive adult patients. The Annals of Thoracic Surgery 75 (2003): 472–478. 12607656 10.1016/s0003-4975(02)04370-9

[77] Oi K, Arai H. Stroke associated with coronary artery bypass grafting. General Thoracic and Cardiovascular Surgery 63 (2015): 487–495. 26153474 10.1007/s11748-015-0572-5

[78] Floyd TF, Shah PN, Price CC, Clinically Silent Cerebral Ischemic Events After Cardiac Surgery: Their Incidence, Regional Vascular Occurrence, and Procedural Dependence. The Annals of Thoracic Surgery: 81 (2006): 2160–2166. 16731147 10.1016/j.athoracsur.2006.01.080

[79] Raffa GM, Agnello F, Occhipinti G, Neurological complications after cardiac surgery: a retrospective case-control study of risk factors and outcome. Journal of Cardiothoracic Surgery 25 (2019). 10.1186/s13019-019-0844-8 PMC6347812 30683130

[80] Hirotani T, Kameda T, Kumamoto T, Stroke after coronary artery bypass grafting in patients with cerebrovascular disease. The Annals of Thoracic Surgery 70 (2000): 1571–1576. 11093489 10.1016/s0003-4975(00)01948-2

[81] U.S. Department of Health & Human Services. Organ Procurement and Transplantation Network. Retrieved from OPTN: Organ Procurement and Transplantation Network.

[82] Ponticelli C, Campise MR. Neurological complications in kidney transplant recipients. J Nephrol 18 (2005): 521–528. 16299677

[83] Ozdemir F, Akgul A, Altunoglu A, The Association Between Cytomegalovirus Infection and Atherosclerotic Events in Renal Transplant Recipients. Transplantation Proceedings 39 (2007): 990–992. 17524871 10.1016/j.transproceed.2007.02.026

[84] Nankivell BJ, Lau SG, Chapman JR, (2000, February). Progression of macrovascular disease after transplantation. Transplantation 69 (2000): 574–581. 10708114 10.1097/00007890-200002270-00019

[85] Adams HP, Dawson G, Coffman TJ, Stroke in Renal Transplant Recipients. Archives of Neurology 43 (1986): 113–115. 3511893 10.1001/archneur.1986.00520020007006

[86] Oliveras A, Roquer J, Puig J, Stroke in renal transplant recipients: epidemiology, predictive risk factors and outcome. Clinical Transplantation 17 (2003): 1–8. 12588314 10.1034/j.1399-0012.2003.02042.x

[87] Ducloux D, Kazory A. Acquired hypercoagulable state in renal transplant recipients. Thrombosis and Haemostasis 91 (2004): 646–654. 15045124 10.1160/TH03-09-0568

[88] Wijdicks EF, Torres VE, Schievink WI, Cerebral Hemorrhage in Recipients of Renal Transplantation. Mayo Clinic Proceedings 74 (1999): 1111–1112. 10560598 10.4065/74.11.1111

[89] Eckhard M, Schindler R, Renner F, New-Onset Diabetes Mellitus After Renal Transplantation. Transplantation Proceedings 41 (2009): 2544–2545. 19715971 10.1016/j.transproceed.2009.06.100

[90] Bodziak KA, Hricik DE. New-onset diabetes mellitus after solid organ transplantation. Transplant International 22 (2008): 519–530. 19040489 10.1111/j.1432-2277.2008.00800.x

[91] Huberlant V, Cosnard G, Hantson PE. Brain Death in a Septic Patient: Possible Relationship with Posterior Reversible Encephalopathy Syndrome? Anaesthesia and Intensive Care 37 (2009): 1017–1020. 20014613 10.1177/0310057X0903700639

[92] Li QS, Huo WQ, Nie ZL, Acute Femoral Neuropathy Following Renal Transplantation: A Retrospective, Multicenter Study in China. Transplantation Proceedings 42 (2010): 1699–1703. 20620504 10.1016/j.transproceed.2010.02.082

[93] United States Renal Data System. 2017 USRDS annual data report: Epidemiology of kidney disease in the United States. National Institutes of Health, National Institute of Diabetes and Digestive and Kidney Diseases, Bethesda, MD (2017).

[94] Ferro CJ, Karim A, Farrugia D, Stroke-Related Hospitalization and Mortality After a Kidney Allograft: A Population-Cohort Study. Exp Clin Transplant 14 (2016): 50–57. 26862824

[95] Du Y, Long Q, Guan B, Robot-Assisted Radical Prostatectomy Is More Beneficial for Prostate Cancer Patients: A System Review and Meta-Analysis. Medical Science Monitor 24 (2018): 272–287. 29332100 10.12659/MSM.907092 PMC5776881

[96] Maerz D, Beck L, Sim A, Complications of robotic-assisted laparoscopic surgery distant from the surgical site. British Journal of Anaesthesia 118 (2017): 492–503. 28403397 10.1093/bja/aex003

[97] Koç G, Tazeh NN, Joudi FN, Lower Extremity Neuropathies After Robot-Assisted Laparoscopic Prostatectomy on a Split-Leg Table. Journal of Endourology 26 (2012): 1026–1029. 22515378 10.1089/end.2011.0653

[98] Wen T, Deibert CM, Siringo FS, Positioning-Related Complications of Minimally Invasive Radical Prostatectomies. Journal of Endourology 28 (2014): 660–667. 24428586 10.1089/end.2013.0623

[99] Gezginci E, Ozkaptan O, Yalcin S, Postoperative pain and neuromuscular complications associated with patient positioning after robotic assisted laparoscopic radical prostatectomy: a retrospective non-placebo and non-randomized study. International Urology and Nephrology 47 (2015): 1635–1641. 26329741 10.1007/s11255-015-1088-8

[100] Song JH, Kaplan JR, Abbott D, Obturator Compartment Syndrome Secondary to Pelvic Hematoma After Robot-Assisted Laparoscopic Radical Prostatectomy. Journal of Endourology Case Reports 2 (2016): 141–143. 27579444 10.1089/cren.2016.0075 PMC4999023

[101] Rajendra Prasad B, Sharma SM, Thomas S, Assessment of shoulder function after functional neck dissection and selective neck dissection (Levels I, II, III) in patients with carcinoma of tongue: a comparative study. Journal of Maxillofacial and Oral Surgery 8 (2009): 224–229. 23139513 10.1007/s12663-009-0055-2 PMC3454242

[102] Balagopal PG, George NA, Sebastian P. Anatomic Variations of the Marginal Mandibular Nerve. Indian Journal of Surgical Oncology 3 (2012): 8–11. 23449987 10.1007/s13193-011-0121-3 PMC3372596

[103] Harada H, Omura K, Takeuchi Y. Patency and caliber of the internal jugular vein after neck dissection. Auris Nasus Larynx 30 (2003): 269–272. 12927290 10.1016/s0385-8146(03)00053-1

[104] Wang L, Liu T, Liu Z. Chronic respiratory dysfunction due to diaphragmatic paralysis following penetrating neck trauma. Medicine 100 (2021): e24043. 33530199 10.1097/MD.0000000000024043 PMC7850730

[105] Gier HHW, Balm JM, Bruning PF, Systematic approach to the treatment of chylous leakage after neck dissection. Head & Neck 18 (1996): 347–351. 8780946 10.1002/(SICI)1097-0347(199607/08)18:4<347::AID-HED6>3.0.CO;2-Y

[106] Teymoortash A, Hoch S, Eivazi B, Postoperative morbidity after different types of selective neck dissection. The Laryngoscope 120 (2010): 924–929. 20222021 10.1002/lary.20894

[107] Quraishi HA, Wax MK, Granke K, Internal Jugular Vein Thrombosis After Functional and Selective Neck Dissection. Archives of Otolaryngology - Head and Neck Surgery 123 (1997): 969–973. 9305248 10.1001/archotol.1997.01900090085012

[108] Smith JS, Shaffrey CI, Fu KMG, Clinical and Radiographic Evaluation of the Adult Spinal Deformity Patient. Neurosurgery Clinics of North America 24 (2013): 143–156. 23561553 10.1016/j.nec.2012.12.009

[109] Schwab F, Dubey A, Gamez L, Adult Scoliosis: Prevalence, SF-36, and Nutritional Parameters in an Elderly Volunteer Population. Spine 30 (2005): 1082–1085. 15864163 10.1097/01.brs.0000160842.43482.cd

[110] Ailon T, Smith JS, Shaffrey CI, Degenerative Spinal Deformity. Neurosurgery 77 (2015): S75–S91. 26378361 10.1227/NEU.0000000000000938

[111] Diebo BG, Passias PG, Marascalchi BJ, Primary Versus Revision Surgery in the Setting of Adult Spinal Deformity. Spine 40 (2015): 1674–1680. 26267823 10.1097/BRS.0000000000001114

[112] Smith JS, Shaffrey CI, Sansur CA, Rates of Infection After Spine Surgery Based on 108,419 Procedures. Spine 36 (2011): 556–563. 21192288 10.1097/BRS.0b013e3181eadd41

[113] Hassanzadeh H, Jain A, El Dafrawy H, Clinical Results and Functional Outcomes of Primary and Revision Spinal Deformity Surgery in Adults. The Journal of Bone and Joint Surgery-American 95 (2013): 1413–1419. 10.2106/JBJS.L.00358 23925747

[114] Smith JS, Ramchandran S, Lafage V, Prospective Multicenter Assessment of Early Complication Rates Associated With Adult Cervical Deformity Surgery in 78 Patients. Neurosurgery 79 (2016): 378–388. 26595429 10.1227/NEU.0000000000001129

[115] He B, Yan L, Xu Z, The causes and treatment strategies for the postoperative complications of occipitocervical fusion: a 316 cases retrospective analysis. European Spine Journal 23 (2014): 1720–1724. 24838504 10.1007/s00586-014-3354-3

[116] Durga P, Sahu B. Neurological deterioration during intubation in cervical spine disorders. Indian Journal of Anaesthesia 58 (2014): 684. 25624530 10.4103/0019-5049.147132 PMC4296351

[117] Lall R, Patel NJ, Resnick DK. A Review of Complications Associated With Craniocervical Fusion Surgery. Neurosurgery 67 (2010): 1396–1403. 20871441 10.1227/NEU.0b013e3181f1ec73

[118] Wright NM, Lauryssen C. Vertebral artery injury in C1-2 transarticular screw fixation: results of a survey of the AANS/CNS Section on Disorders of the Spine and Peripheral Nerves. Journal of Neurosurgery 88 (1998): 634–640. 9525707 10.3171/jns.1998.88.4.0634

[119] Jung A, Schramm J. How to Reduce Recurrent Laryngeal Nerve Palsy in Anterior Cervical Spine Surgery. Neurosurgery 67 (2010): 10–15. 20559087 10.1227/01.NEU.0000370203.26164.24

[120] Yasuda T, Togawa D, Hasegawa T, Hypoglossal Nerve Palsy as a Complication of an Anterior Approach for Cervical Spine Surgery. Asian Spine Journal 9 (2015): 295. 25901245 10.4184/asj.2015.9.2.295 PMC4404548

[121] Civelek E, Karasu A, Cansever T, Surgical anatomy of the cervical sympathetic trunk during anterolateral approach to cervical spine. European Spine Journal 17 (2008): 991–995. 18548289 10.1007/s00586-008-0696-8 PMC2518767

[122] Adams HD, Van Geertruyden HH. Neurologic Complications of Aortic Surgery. Annals of Surgery 144 (1956): 574–610. 13373248 10.1097/00000658-195610000-00005 PMC1465537

[123] Riambau V, Böckler D, Brunkwall J, Editor’s Choice- Management of Descending Thoracic Aorta Diseases. European Journal of Vascular and Endovascular Surgery 53 (2017): 4–52. 28081802 10.1016/j.ejvs.2016.06.005

